# Food-induced anaphylaxis and cofactors – data from the anaphylaxis registry 

**DOI:** 10.5414/ALX01401E

**Published:** 2017-08-04

**Authors:** M. Worm, K. Scherer, A. Köhli-Wiesner, F. Ruëff, V. Mahler, L. Lange, R. Treudler, E. Rietschel, Z. Szepfalusi, R. Lang, U. Rabe, T. Reese, N. Schwerk, K. Beyer, S. Hompes, A. Bircher, B. Przybilla, T. Hawranek, G. Hansen, F. Friedrichs, H. Merk, K. Tenbrock, S. Lehmann, M. Gerstlauer, J. Kleine-Tebbe, B. Niggemann, H. Dickel, M. Bücheler, T. Bieber, J. Hanfland, S. Schmitt-Grohe, D. Vlajnic, V. Heckmann, K. Nemat, K. Schäkel, A. Nordwig, A. Schuster, S. Schweitzer-Krantz, U. Hillen, M. Kopp, C. Szliska, J. Klinge, I. Neustädter, T. Fuchs, R. Bruns, C. Marsch, B. Kreft, E. Coors, W. Rebien, B. Wedi, C. Pföhler, M. Rett, M. Henzgen, P. Vöhringer, R. Fölster-Holst, N. Hunzelmann, G. Siebenhaar, S. Nestoris, C. Schirpke, J. Grabbe, G. Stichtenoth, J. Ring, K. Brockow, R. Brehler, I. Yildiz, S. Volkmuth, M. Geißler, M. Polz, F. Riffelmann, S. Thies, U. Lepp, U. Rabe, H. Rebmann, T. Spindler, L. Klimek, O. Pfaar, W. Brosi, W. Aberer, E. Varga, N. Reider, I. Huttegger, T. Kinaciyan, K. Hoffmann-Sommergruber, P. Eng, A. Helbling, P. Eigenmann, R. Guggenheim, P. Schmid-Grendelmeier

**Affiliations:** 1 Klinik für Dermatologie und Allergologie, Charité – Universitätsmedizin Berlin; 2 Abteilung Allergologie, Klinik für Dermatologie, Universitätsspital Basel, Schweiz; 3 Allergologie, Universitätskinderkliniken Zürich, Schweiz; 4 Klinik und Poliklinik für Dermatologie und Allergologie, Ludwig-Maximilians-Universität München; 5 Hautklinik Universitätsklinikum Erlangen; 6 Abteilung für Pädiatrie, St.-Marien-Hospital Bonn; 7 Klinik für Dermatologie, Venerologie und Allergologie, Universität Leipzig; 8 Klinik und Poliklinik für Kinderheilkunde, Uni-Klinik Köln; 9 Klinik für Kinder und Jugendheilkunde, Medizinische Universität Wien, Österreich; 10 Klinik für Dermatologie, Paracelsus Medizinische Privatuniversität Salzburg, Österreich; 11 Fachklinik für Pneumologie, Johanniter-Krankenhaus, Treuenbrietzen; 12 Klinik für Kinder- und Jugendmedizin, Mathias-Spital Rheine; 13 Zentrum für Kinderheilkunde und Jugendmedizin, Medizinische Hochschule Hannover; 14 Klinik für Pädiatrie mit Schwerpunkt Pneumologie und Immunologie, Charité – Universitätsmedizin Berlin; 15 Kinderarztpraxis Laurensberg Aachen; 16 Klinik für Dermatologie, Universitätsklinik Aachen RWTH; 17 Universitätskinderklinik Aachen, RWTH; 18 Klinik für Kinder und Jugendliche am Klinikum Augsburg; 19 Allergie- und Asthma-Zentrum Westend, Berlin; 20 Pädiatrische Allergologie und Pneumologie, DRK-Kliniken Berlin I Westend; 21 Klinik für Dermatologie, Ruhr-Universität Bochum; 22 HNO-Abteilung, Ev. Kliniken Bonn; 23 Klinik für Dermatologie, Universitätsklinik Bonn; 24 Zentrum für Kinderheilkunde, Universitätsklinik Bonn; 25 Prof. Hess Kinderklinik, Klinikum Bremen; 26 Klinik für Jugendmedizin, Universitätsklinikum TU, Dresden; 27 7Klinik für Dermatologie, Universitätsklinikum TU, Dresden; 28 Zentrum für Kinder- und Jugendmedizin, Städtisches Krankenhaus Dresden; 29 Klinik für Kinderkardiologie und Pneumologie, Universitätsklinikum Düsseldorf; 30 Klinik für Kinder- und Jugendmedizin, Ev. Krankenhaus Düsseldorf; 31 Hautklinik, Universitätsklinik Essen; 32 Zentrum für Kinder- und Jugendmedizin, Albert-Ludwigs-Universität Freiburg; 33 Klinik für Dermatologie, Krankenhaus Bethesda, Freudenberg; 34 Klinik für Kinder- und Jugendmedizin, Klinikum Fürth; 35 Klinik für Dermatologie, Georg-August-Universität Göttingen; 36 Zentrum für Kinder- und Jugendmedizin, Ernst-Moritz-Arndt-Universität Greifswald; 37 Klinik für Dermatologie, M.-L.-Universität Halle-Wittenberg; 38 Klinik für Dermatologie, Universitätsklinikum Hamburg; 39 Allergologische Schwerpunktpraxis Rebien Hamburg; 40 Klinik für Dermatologie, Medizinische Hochschule Hannover; 41 Klinik für Dermatologie und Allergologie, Universitätsklinik des Saarlandes, Homburg; 42 Klinik für Kinder- und Jugendmedizin, Klinikum Itzehoe; 43 Pneumologie/Allergologie der Klinik für Innere Medizin I, Friedrich-Schiller-Universität Jena; 44 Klinik für Kinder- und Jugendmedizin, Städtisches Klinikum Karlsruhe; 45 Hautklinik des Universitätsklinikums Kiel; 46 Hautklinik des Universitätsklinikums Köln; 47 Klinik für Dermatologie, Klinikum Lippe-Lemgo; 48 Zentrum für Pneumologie, Lungenklinik Lostau; 49 Klinik für Dermatologie und Allergologie, Universitätsklinikum Lübeck; 50 Klinik für Kinder- und Jugendmedizin, Universitätsklinikum Lübeck; 51 Klinik für Dermatologie, Technische Universität München; 52 Klinik für Hautkrankheiten, Universitätsklinikum Münster; 53 Klinik für Jugendmedizin, Friedrich-Ebert-Krankenhaus Neumünster; 54 Abteilung für Kinder- und Jugendmedizin, Klinikum Niederberg Velbert; 55 HNO-Klinik Otto-Körner, Rostock; 56 Klinik für Kinder- und Jugendmedizin, Rüsselsheim; 57 Abteilung Allergologie, Fachkrankenhaus Kloster Grafschaft/Schmallenberg; 58 Hautklinik, Asklepios Klinikum Uckermark Schwedt; 59 Herz-Lungen-Praxis Stade; 60 Fachklinik für Pneumologie, Johanniter-Krankenhaus, Treuenbrietzen; 61 Klinik für Kinder- und Jugendmedizin, Universitätsklinikum Tübingen; 62 Waldburg-Zeil-Kliniken, Kinder- und Jugendmedizin, Fachkliniken Wangen im Allgäu; 63 Zentrum für Rhinologie, Universitätsklinikum Wiesbaden; 64 Kinderpneumologie, -allergologie, Ärzte- und Bürohaus am Dom, Würzburg; 65 Universitäts-Hautklinik Graz, Österreich; 66 Universitätsklinik für Kinder- und Jugendheilkunde Graz, Österreich; 67 Klinik für Dermatologie, Medizinische Universität Innsbruck, Österreich; 68 Landesklinik für Jugendheilkunde, Salzburg, Österreich; 69 Klinik für Dermatologie und Zentrum für Physiologie und Pathophysiologie, Medizinische Universität Wien, Österreich; 70 Pädiatrische Allergologie und Pneumologie, Kinderklinik Aarau, Schweiz; 71 Allergologische Poliklinik, Universitätsspital Bern, Schweiz; 72 Pädiatrische Klinik, Universitätsspital Genf, Schweiz; 73 Pädiatrische Allergologie und Pneumologie, Kinderspital Luzern, Schweiz; 74 Klinik für Kinder- und Jugendmedizin, Stadtspital Triemli Zürich, Schweiz; 75 Dermatologische Klinik, Universitätsspital Zürich, Schweiz

**Keywords:** anaphylaxis, registry, food allergens, cofactors

## Abstract

Food allergens are frequent causes of anaphylaxis. In particular in children and adolescents they are the most frequent elicitors of severe allergic reactions, and in adults food allergens rank third behind insect venom and drugs. Since July 2006 severe allergic reactions from Germany, Austria, and Switzerland are collected in the anaphylaxis registry. Currently 78 hospitals and private practises are connected. From July 2006 until February 2009 1,156 severe allergic reactions were registered. Among children and adolescents (n = 187, age range from 3 months to 17 years) food allergens were the most frequent triggers, comprising 58% of cases. In the adult group (n = 968, 18 – 85 years) food allergens were in the third position (16.3%) behind insect venom and drugs. In children legumes (31%) and in particular peanuts were frequently responsible food allergens, followed by tree nuts (25%) with hazelnut being the most frequent elicitor. In adults fruits (13.4%) most often induced severe food-dependent anaphylaxis, but also animal products (12.2%); among these most frequently crustaceans and molluscs. Cofactors were often suspected in food-dependent anaphylaxis, namely in 39% of the adult group and in 14% of the pediatric group. In adults drugs (22%) and physical activity (10%) were reported to be the most frequent cofactors, in children physical activity was suspected in 8.7% and drugs in 2.6%. Concomitant diseases like atopic dermatitis, allergic asthma, or allergic rhinoconjunctivitis were reported in 78% of children and adolescents and in 67% of the adults. In conclusion, food-induced anaphylaxis, its cofactors and concomitant diseases are age-dependent. The data offers to identify risk factors of anaphylaxis.

German version published in Allergologie, Vol. 34, No. 7/2011, pp. 329-337

## Introduction 

The anaphylaxis registry includes patients who have experienced a severe allergic reaction with involvement of the respiratory tract and/or the cardiovascular system. Data is collected via a password-protected online questionnaire from allergy centers in Germany, Austria, and Switzerland. Demographic patient data, triggers of the reaction as well as data on concomitant diseases and the presence of other possible cofactors, like physical activity and intake of drugs, are registered. Food has been reported to be a frequent cause of severe allergic reactions [1, 2]. Earlier reports analyzing hospital-based data of anaphylaxis patients show that 25 – 60% of anaphylactic reactions were caused by food [3]. These early data is supported and confirmed by more recent systematic and epidemiologic investigation. Among foods mainly peanuts and tree nuts are frequently responsible for the induction of severe allergic reactions and even for fatalities [4, 5, 6]. 

While numerous studies are available for the inducing factors of severe allergic reactions, the data on the impact of cofactors is very limited. Some publications suggest that physical activity and the intake of certain drugs can influence the occurrence or the severity of allergic reactions [7]. The cofactor physical activity has, for instance, been described for certain food allergens like wheat or shellfish, but also for other types of food [8, 9, 10]. 

Current data is mainly based on single case reports or case series, while systematic data on the impact of cofactors is missing. Thus, we aimed at investigating the presence and frequency of possible cofactors in cases of food-induced anaphylaxis. 

## Material and methods 

We evaluated data from a total of 1,165 anaphylactic reactions that were included in the anaphylaxis registry between July 2006 and February 2009. Only data of patients who had experienced a severe allergic reaction with airway and/or cardiovascular involvement were taken into account [11]. 

## Results 

Of the 1,165 anaphylactic reactions 271 were induced by food. Of these, 115 (42.4%) events took place in the group of children and adolescents (age 3 months to 17 years) and 156 (57.6%) cases in the adult group (age 18 – 85 years). In the group of children and adolescents food was the most frequent cause of severe allergic reactions (58%); in adults food allergens (16%) ranked third behind insect venom (55%) and drugs (21%). 

### Peanuts are the most frequent triggers of severe allergic reactions in children

For children and adolescents the detailed analysis for groups of food demonstrated that legumes (31.3%) are the most frequent triggers, followed by tree nuts (25.2%) and various animal products (22.6%) (Table 1). Further food groups like fruits, spices, vegetables, and cereals induced severe allergic reactions in less than 5% of cases. 

### Crustaceans and shellfish are very frequently reported to induce severe allergic reactions in adults

The detailed evaluation of foods as triggers of severe allergic reactions in adults showed a different profile than that for children and adolescents (Table 1). The most frequent triggers came from the category of fruits, followed by animal products. Among the animal products the most frequently reported allergen sources were crustaceans and shellfish (n = 11), thus representing the most frequently reported (in absolute numbers) foods inducing severe allergic reactions in adults. The food group of vegetables is as frequently reported to induce severe allergic reactions in adults, with celery (n = 8) and carrot (n = 7) being the most frequent sources. In adults combinations of foods were more frequently reported (n = 18), while in the pediatric group combinations were reported in only 4.3% of cases. In contrast to the pediatric patients, the group of legumes ranked fourth in the adults, with soy – rather than peanut – being the most frequent trigger (n = 8). 

### Cofactors – analysis of the reported food-induced anaphylactic reactions 

**Figure 1.**
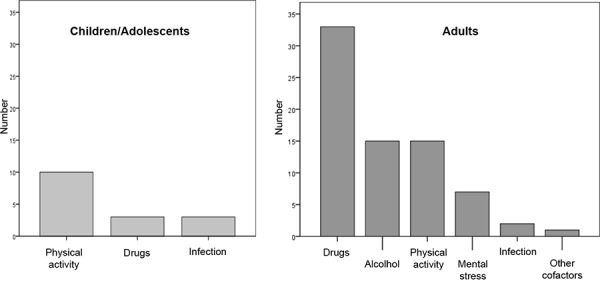
Cofactors in children/adolescents (n = 16) and adults (n = 60) when foods are indicated as triggers of allergic reactions.

In the children and adolescent group the analysis of the frequency of cofactors shows that cofactors were reported in 14% of cases; the most frequent one was physical activity (Figure 1). Infections and drugs were only rarely indicated to be cofactors. In the adult group cofactors were reported markedly more often (n = 60; 39%) (Figure 1). The most frequently indicated cofactor was drug intake, followed by alcohol and increased physical and mental stress. Infections were indicated in 2 cases in the adult group. The most frequently reported drugs were beta-blockers (n = 10), acetylsalicylic acid (n = 9), other analgesics (n = 6) and ACE-inhibitors (n = 6). 

### Comorbidities in patients with food-induced severe allergic reactions 

**Figure 2:**
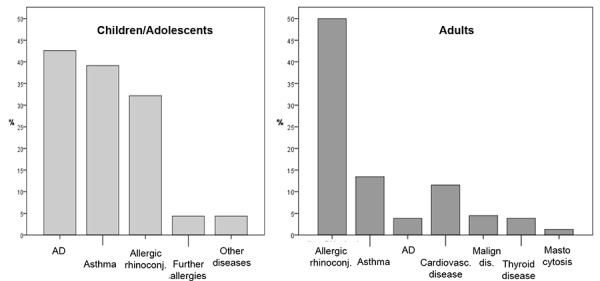
Figure 2. Frequency of underlying diseases in children/adolescents (n = 115) and adults (n = 156).

In children and adolescents who indicated foods as triggers of severe allergic reactions, the most frequently reported concomitant underlying diseases were atopic ones (Figure 2). Most frequently atopic dermatitis was indicated (43%), followed by allergic asthma (39%) and allergic rhinoconjunctivitis (32%). In adults atopic diseases were also frequently reported as concomitant underlying disease, with allergic rhinoconjunctivitis (50%), allergic asthma (14%), and, less frequently, atopic dermatitis (4%) were indicated (Figure 2). Further registered comorbidities were cardiovascular diseases (12%), while mastocytosis was present in only 2 cases (1%). In adults, the comparative analysis of underlying diseases between food-induced and non-food-induced cases showed that in non-food-induced severe allergic reactions cardiovascular diseases were predominant (25%). 

## Discussion

Our data show that the registered cases in the anaphylaxis registry can provide detailed and age-based information on the triggers or groups of triggers of severe allergic reactions and, in addition, can allow for the assessment of concomitant circumstances and comorbidities. Data from this type of clinical epidemiologic investigation allows to identify risk factors for the occurrence of severe allergic reactions in the future. 

Our results concur with those published in the literature and show that food is the most frequent trigger of severe allergic reactions in children and adolescents [11, 12]. In adults food also plays an important role, but only ranks third in frequency after insect venom and drugs. The most frequently reported inducing food in children and adolescents was peanut. Peanut is a well-known food allergen, particularly for inducing severe reactions after contact with relatively low amounts. It was surprising, however, that this allergen was registered so frequently in Germany, Austria, and Switzerland. Until recently, peanuts were considered to be an important food allergen mainly in the UK and USA. Unlike, for example, infants with milk allergy, patients with peanut allergy only rarely develop natural tolerance and therefore it has to be expected that – if no efficient therapies are developed – in the next decades more and more adults will experience severe allergic reactions caused by peanuts. 

The anaphylaxis registry aims at displaying these developments and at the same time improving the therapy concepts for affected patients. Further important and frequent food allergens that cause severe allergic reactions in children and adolescents were hazelnut and cow’s milk. This data also is in accordance with preceding epidemiologic data that show that these allergens play an important role in children and adolescents [15]. In adults the profile of triggers shows frequent sorts of fruits as well as crustaceans and shellfish to induce severe allergic reactions. Crustaceans and shellfish are currently being indicated in accordance with the labeling obligation. For further investigation it would be interesting to know whether the patients ingested these types of food inadvertently or due to limitations of the labeling obligation (e.g., ingestion of unpacked products). Another frequently reported group of triggers in adults is vegetable, in particular celery and carrot. As celery contains heat-stable food allergens and as it is also subject to labeling obligation, it would be important to document the exact circumstances including the amount of ingested food. 

The data on the presence of cofactors demonstrate that cofactors were more frequently reported for severe allergic reactions in adults and that the profile of these cofactors was age-dependent. In children and adolescents the most frequently reported cofactors were physical activity and infections, while in adults drugs, alcohol consumption, and physical activity were indicated most frequently. Currently, little is known about the pathophysiologic mechanisms by which cofactors can influence the induction and the severity of severe allergic reactions. Presumably they are of importance more frequently than indicated; however, this must be observed more closely in the future. Patients should be better informed about the importance of cofactors as this will lead to more detailed information on their presence. So far, prospective controlled clinical studies are lacking. In the literature mainly case reports or case series are available [9, 16, 17]. 

Another interesting aspect is the observation that drugs like beta-blockers, analgesics, and ACE inhibitors were reported as being cofactors for the development of severe allergic reactions. In this context it has to be taken into account that due to the high prevalence of cardiovascular diseases these kinds of drugs are very frequently used in the general population. Therefore, a control group analysis would be necessary to find out whether a relative accumulation of the use of these drugs is present in the affected adult group. On the other hand, at least drugs like analgesics, but also ACE inhibitors, seem to promote the induction or the severity of mast cell-dependent reactions so that it might be possible that these types of drugs are important in the context of severe allergic reactions or, alternatively, that they are indicated more frequently by allergists. And this is were data acquisition reaches its limitations: The indication of a possibly important cofactor is usually based on patient history and not on repeated challenge tests with and without this cofactor. 

Data on concomitant diseases shows that allergic diseases were very frequent in affected children and adolescents. This is in accordance with data on the epidemiology of allergic diseases in childhood. For the group of foods these observation suggest that children with food allergy and further allergic reasons have an increased anaphylaxis risk. The anaphylaxis registry also allows for a prospective identification of patients with repeated reactions, thus displaying the course of the disease “anaphylaxis”. Also for adults data shows a high prevalence of allergic diseases, with allergic rhinoconjunctivitis – in accordance with its epidemiology – being the most frequently reported one. Interestingly, the comparison with non-food-dependent severe allergic reactions showed that cardiovascular diseases were less frequent and the triggers were primarily pollen-associated foods. Nevertheless, the group of shellfish and crustaceans could be identified as a further frequent food allergen related to severe allergic reactions in adults. Further investigation considering the exact sensitization profile will possibly allow to better differentiate between primary and secondary food allergies and thus to provide better preventive and therapeutic measures. 

## Summary 

In conclusion, our data demonstrate that food frequently induces severe allergic reactions in adults and particularly in children, with inhalant allergy and/or atopic dermatitis being probable risk factors. The data provides important information for the future detection of risk profiles for affected patients and facilitate the identification of foods that can induce severe allergic reactions. 

**Table 1.** The most frequently reported foods inducing anaphylaxis in children/adolescents and adults.

**Food groups**	**Foods**	**Children**	**Adults**
		**n**	**n**
**Legumes**	Peanut	26	4
	Soy	1	8
	Pea	2	0
	Chickpea	1	0
	Lupin	1	1
	*Non-specified legumes*	5	4
	** n** (total)	**36**	**17**
**Tree nuts**	Hazelnut	11	5
	Cashew nut	5	1
	Almond	4	3
	Walnut	3	3
	Pistachio	2	1
	Pine nut	0	2
	Brazi nut	1	1
	*Non-specified nuts*	3	1
	** n** (total)	**29**	**17**
**Animal products**	Cow’s milk	11	0
	Hen’s egg	8	2
	Fish	4	2
	Crustaceans and shellfish	2	11
	Pork	0	1
	*Non-specified animal products*	1	3
	** n** (total)	**26**	**19**
**Fruits**	Appel	2	2
	Mango	2	2
	Banana	0	2
	Date	0	2
	Lychee	1	1
	Fig, blueberry, kiwi	0	1 each
	Peach, plum, gooseberry	0	1 each
	Grape, citrus fruit	0	1 each
	Non-specified fruits	0	4
	** n** (total)	**5**	**21**
**Vegetable**	Celery	2	8
	Carrot	0	7
	Tomatoe	0	2
	Bell pepper	1	0
	Chikory, parsnip	0	1 each
	** n** (total)	**3**	**19**
